# Circadian rhythms in metabolic organs and the microbiota during acute fasting in mice

**DOI:** 10.14814/phy2.15393

**Published:** 2022-07-19

**Authors:** Lauren Pickel, Ju Hee Lee, Heather Maughan, Irisa Qianwen Shi, Navkiran Verma, Christy Yeung, David Guttman, Hoon‐Ki Sung

**Affiliations:** ^1^ Translational Medicine Program, The Hospital for Sick Children Toronto Ontario USA; ^2^ Department of Laboratory Medicine and Pathology University of Toronto Toronto Ontario USA; ^3^ Ronin Institute Montclair New Jersey USA; ^4^ Centre for the Analysis of Genome Evolution & Function University of Toronto Toronto Ontario USA

## Abstract

The circadian clock regulates metabolism in anticipation of regular changes in the environment. It is found throughout the body, including in key metabolic organs such as the liver, adipose tissues, and intestine, where the timing of the clock is set largely by nutrient signaling. However, the circadian clocks of these tissues during the fasted state have not been completely characterized. Moreover, the sufficiency of a functioning host clock to produce diurnal rhythms in the composition of the microbiome in fasted animals has not been explored. To this end, mice were fasted 24 h prior to collection of key metabolic tissues and fecal samples for the analysis of circadian clock gene expression and microbiome composition. Rhythm characteristics were determined using CircaCompare software. We identify tissue‐specific changes to circadian clock rhythms upon fasting, particularly in the brown adipose tissue, and for the first time demonstrate the rhythmicity of the microbiome in fasted animals.

## Graphical abstract

Fasting causes tissue‐dependent changes in core clock gene expression, with the core clock of BAT being most sensitive to fasting. Variation in the microbiome depends more on circadian time than on feeding or fasting condition.

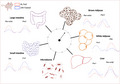

## 1 INTRODUCTION

The circadian clock is a transcription‐translation feedback loop found ubiquitously in all mammalian cells (Takahashi, 2017). The clock regulates metabolic processes in anticipation of daily rhythms in energy availability and demand. The fidelity of this prediction is maintained by adjustments of the clock (entrainment) to environmental cues (Zeitgebers). While the central hypothalamic clock in the suprachiasmatic nucleus (SCN) responds primarily to light as a Zeitgeber, feeding and fasting are the key Zeitgebers for metabolic tissues (Pickel & Sung, 2020). For example, time‐restricted feeding can cause a complete phase inversion of peripheral tissue clocks, independent of the light cycle and SCN entrainment (Damiola et al., 2000; Hara et al., 2001; Stokkan et al., 2001). Peripheral clock entrainment occurs largely through adjustment of clock gene expression in response to signals of energy availability. For example, peripheral tissue clocks are responsive to fasting‐associated signals of circulating ghrelin (Wang et al., 2018) and glucagon (Sun et al., 2015) and intracellular activation of AMPK, PCG1a, and SIRT1 (Froy & Garaulet, 2018), as well as to postprandial increases in circulating insulin and IGF‐1, and intracellular mTORC1 activation (Crosby et al., 2019; Lipton et al., 2015).

Importantly, adjustments of the core clock orchestrate a cascade of downstream changes in the transcriptome and metabolome (Panda, 2016). The clocks of different tissues and the processes they regulate can become misaligned in time when exposure to key Zeitgebers is discrepant, for example, when humans and other diurnal mammals eat during the dark (inactive) phase. Inconsistencies in the timing of Zeitgeber exposure can also produce misalignment because the entrainment of the core clock to nutrient signaling occurs at different rates in peripheral tissues (Damiola et al., 2000). This is important because circadian clock misalignment is strongly associated with metabolic diseases (James et al., 2017; Kervezee et al., 2020; Kolbe et al., 2019; Scheer et al., 2009). On the other hand, improving circadian alignment through time‐restricted feeding protects metabolic health (Chaix et al., 2014, 2021; Jamshed et al., 2019).

The functions of major metabolic organs, including the liver, white and brown adipose tissues, skeletal muscle, and intestine are highly dependent on whether an animal is in the fed or fasted state (Secor & Carey, 2016). Processes of nutrient absorption, macronutrient catabolism and energy storage are altered in response to, and in anticipation of, the fasting state, and this anticipatory regulation depends on the local circadian clock of peripheral tissues (Lamia et al., 2008). Therefore, the response of the core clock in these metabolic organs to fasting is of great interest. However, while the rhythmic expression of core clock genes in these metabolic tissues has been studied in ad libitum (AL) fed animals, the circadian clock of fasting animals has only been characterized in the liver and skeletal muscle (Kinouchi et al., 2018; Shavlakadze et al., 2013). The regulation of core clock genes in the gut and adipose tissue of fasted animals remains unknown despite these being two critical loci of fasting physiology. In addition, previous investigations of the clock's response to acute fasting in metabolic organs confounded the variables of circadian time and fasting duration by beginning the fast of all animals simultaneously (Kawamoto et al., 2006; Sun et al., 2015). This distinction is essential for meaningful interpretation, given that peripheral tissue clocks are sensitive to the duration of fasting (Kuroda et al., 2012).

Moreover, the circadian response of the microbiome to fasting has not been investigated. The microbiome affects systemic metabolism and energy status through production of bacterial metabolites and by modulating nutrient absorption in the intestine. The composition and function of the gut microbiota varies with a daily rhythm (Liang et al., 2015; Thaiss et al., 2014, 2016), and similar to clocks in other peripheral tissues, a robust rhythmicity is indicative of health; disrupted microbial rhythms predicted T2D in large human cohorts (Reitmeier et al., 2020). However, the circadian profile of the microbiome has not been characterized in fasted animals. It is unknown whether its rhythms are entirely dependent on rhythmic nutrient availability or can persist during fasting.

We therefore sought to characterize the multi‐organ circadian response to fasting. Gene expression analysis was performed for all core clock genes in the liver, brown and white adipose tissue, duodenum, and colon at 4‐h intervals in 24‐h fasted mice. To test the sufficiency of the core clock to produce rhythms in the microbiota in the absence of food intake, 16S rRNA sequencing was performed on fecal samples collected from animals as they were sacrificed for tissue collection.

## 2 METHODS

### 2.1 Animals

All animal experimental protocols approved by the Animal Care Committee of the Centre of Phenogenomics (TCP) conformed to the standards of the Canadian Council on Animal Care. Eight to 10‐week‐old male C57BL/6J mice were housed under a 12:12 light–dark cycle with AL access to normal chow diet (Teklad Global #2918) and water. Food was withheld for the fasting group beginning 24 h prior to the respective sacrifice time. Fasted and ad‐libitum fed mice were sacrificed at 4 h intervals over a 24 h period beginning at ZT0 (6 time points, *n* = 3 mice per time point per group). Mice of the same time point and feeding condition were housed together in solid bottomed cages in the days leading up to the experiment. Tissues were collected and flash frozen in liquid nitrogen. Feces were removed from the distal intestine, and intestine was rinsed in PBS. Sections of colon and duodenum were resected immediately distal to the cecum and to the stomach, respectively.

### 2.2 RNA extraction and reverse transcription

Tissues were separately homogenized in TRIzol and total RNA was extracted from liver, perigonadal white adipose tissue (PWAT), interscapular brown adipose tissue (BAT), duodenum and colon using an RNeasy Mini Kit (Qiagen). Complimentary DNA (cDNA) was synthesized by reverse transcription of RNA. DNA was extracted from stool using NucleoSpin Soil Mini Kit (Macherey Nagel).

### 2.3 Quantitative real‐time PCR

Quantitative real‐time PCR (RT‐qPCR) was performed on cDNA using SYBR Green Master Mix (ThermoFisher) and QuantStudio Real‐Time PCR System. Primer sequences are listed in Table S1.

### 2.4 16S rRNA gene sequencing

16S rRNA gene sequencing was performed by The Centre for the Analysis of Genome Evolution and Function (CAGEF) at the University of Toronto. The V4 hypervariable region of the 16S rRNA gene was amplified using uniquely barcoded 515F (forward) and 806R (reverse) sequencing primers to allow for multiplexing (Caporaso et al., 2012). Amplification reactions were performed using 12.5 μl of KAPA2G Robust HotStart ReadyMix (KAPA Biosystems), 1.5 μl of 10 μM forward and reverse primers, 7.5 μl of sterile water and 2 μl of DNA. The V4 region was amplified by cycling the reaction at 95°C for 3 min, 18x cycles of 95°C for 15 s, 50°C for 15 s and 72°C for 15 s, followed by a 5‐min 72°C extension. All amplification reactions were done in duplicate to reduce amplification bias, pooled, and checked on a 1% agarose TBE gel. Pooled duplicates were quantified using PicoGreen and combined by even concentrations. The library was then purified using Ampure XP beads and loaded on to the Illumina MiSeq for sequencing, according to manufacturer instructions (Illumina, San Diego, CA). Sequencing is performed using the V2 (150bp x 2) chemistry.

### 2.5 Analysis of the bacterial microbiome

The UNOISE pipeline, available through USEARCH v11.0.667 and vsearch v2.10.4, was used for sequence analysis (Edgar, 2010, 2013, 2016; Rognes et al., 2016). Sequences were assembled and quality trimmed using –fastq_mergepairs with a –fastq_trunctail set at 2, a –fastq_minqual set at 3, a ‐fastq_maxdiffs set at 5, a ‐fastq_pctid set at 90, and minimum and maximum assemble lengths set at 243 and 263 (+/− 10 from the mean) base pairs. Assembled sequences were quality filtered using –fastq_filter with a –fastq_maxee set at 1.0. Sequences were de‐replicated and sorted to remove singletons, then denoised, and chimeras were removed using the unoise3 command. Assembled sequences were mapped back to the chimera‐free denoised sequences at 99% identity operational taxonomic units (OTUs), which are units of diversity that approximate groups of bacterial species or strains.

QIIME2 v2021.4 (Bolyen et al., 2019) was used for the following analyses. Taxonomy was assigned to each OTU via q2‐feature‐classifier (Bokulich et al., 2018) with the classify‐sklearn naïve Bayesian classifier and the Greengenes 13_8 99% OTUs reference set based on the 515F/806R primer region (McDonald et al., 2012). Abundances of taxa were plotted using data that were raw (relative abundances) or rarefied (count abundances) to the lowest per sample sequence count (34,800 reads). ANCOM analyses (Mandal et al., 2015) were performed via q2‐composition using OTU tables to which a pseudocount of ‘1’ had been added to remove zeros.

MicrobiomeAnalyst (Chong et al., 2020; Dhariwal et al., 2017) was used for the following analyses. LEfSe (Segata et al., 2011) was performed on raw genus‐level data using a false discovery rate (Benjamini & Hochberg, 1995) cutoff of 0.1 and a LDA score cutoff of 2.0. Alpha diversity was estimated using Shannon and Simpson indices. Beta diversities, estimated using Bray‐Curtis dissimilarities (Bray & Curtis, 1957), were plotted in multivariate space using principal coordinates analysis. PERMANOVA (Anderson, 2001) was used to identify significant differences between sample beta diversities grouped by timepoint or feeding. For diversity analyses the data were normalized, unless otherwise indicated, by removing OTUs in fewer than 4 samples and whose interquartile range varied by less than 10%; total‐sum scaling was also performed.

### 2.6 Statistical analysis

RT‐qPCR data were normalized to expression of *36B4* in all tissues, and to the geometric mean of *36B4* and *Hmbs* in BAT to account for minor rhythmicity of individual housekeeping genes (Figure S1a). Data are presented as means + SEM and plotted relative to the ZT0 Fed condition. A 2‐way repeated‐measures ANOVA with post‐hoc Bonferroni was performed (SPSS) using the mean of technical replicates to test for effects of time, feeding group, and their interaction. Differences in expression at single time points were identified with a Student's TTEST. Periodicity and changes in amplitude, mesor (rhythm‐adjusted mean), and phase in the fasted compared to fed condition were analyzed in the R package CircaCompare; for genes rhythmic in only one condition, these parameters were estimated using circa_single (Parsons et al., 2020).

## 3 RESULTS

### 3.1 Fasting alters core clock rhythms in a tissue‐specific manner

**FIGURE 1:**
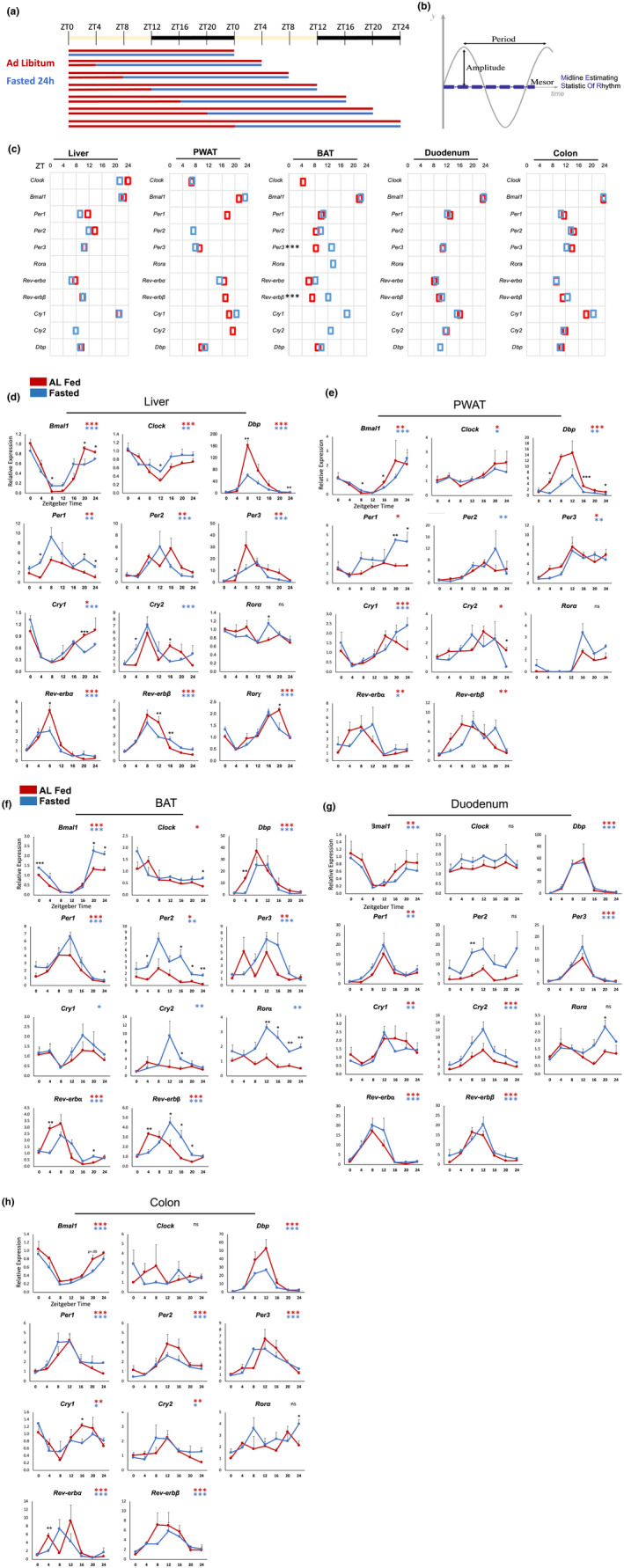
Core clock rhythms in key metabolic organs of 24 h fasted mice. (a) Fasting schedule. (b) the three primary characteristics of rhythms: Period, amplitude, and Mesor—A rhythm‐adjusted mean expression level (MESOR, midline estimating statistic of rhythm). (c) Acrophase (time of peak expression) of significantly rhythmic core clock genes in AL fed (red) and 24 h fasted (blue) mice. (d–f) expression of core clock genes in liver, PWAT, BAT, duodenum, and colon. Data plotted relative to ZT0 fed as mean + SEM (*n* = 3 replicates per time point per group). Black asterisks indicate significant differences between fed and fasted groups at a particular timepoint (TTEST), *n* = 3 mice/timepoint/group. Colored asterisks indicate presence of 24 h rhythm. *p < 0.05, **p < 0.01, ***p < 0.001.

**FIGURE 2:**
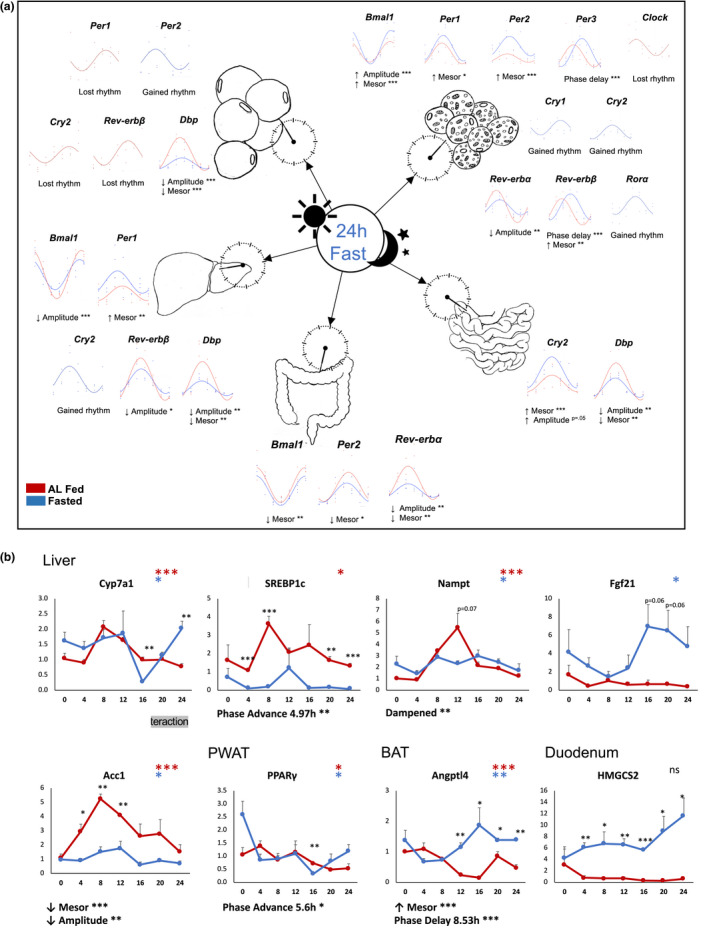
An acute fast alters core clock and metabolic gene expression rhythms. (a) Comparison of rhythm characteristics in AL fed (red) and 24 h fasted (blue) mice, analyzed by Circacompare. (b) Expression of clock‐controlled genes. Black asterisks indicate significant differences between fed and fasted groups at a particular timepoint (TTEST), *n* = 3 mice/timepoint/group. Colored asterisks indicate presence of 24 h rhythm. *p < 0.05, **p < 0.01, ***p < 0.001.

Mice were fed ad libitum or fasted for 24 h prior to the respective sacrifice time (Figure 1a). A constant fasting duration allows isolation of the circadian time as the sole variable. The rhythmicity of core clock genes and their rhythm characteristics of amplitude, period, and mesor (rhythm‐adjusted mean) (Figure 1B) were analyzed using CircaCompare (Parsons et al., 2020). The mRNA expression rhythms of core clock genes in the liver of fed and 24 h fasted mice were consistent with those observed in previous work (Kinouchi et al., 2018). With this experimental design and methods, we further analyzed the expression of core clock genes in the perigonadal white adipose tissue (PWAT), brown adipose tissue (BAT), duodenum, and colon of AL fed and 24 h fasted animals. Fed state rhythms replicated previous results in the adipose (Zvonic et al., 2006), gut (Polidarová et al., 2009) and liver (Storch et al., 2002). Significant phase shifts (Figure 1c) were only observed in BAT, where expression of *Per3* and *Rev‐erbβ* were phase delayed (4.87 and 5.01 h, respectively, *p* < 0.001). A trend toward phase delay was also observed in *Cry1* in the PWAT (2.27 h, *p* = 0.06). Nearly all core clock genes were rhythmic in both the fed and fasted conditions (Figure 1d–h), though some rhythms were lost and gained, or rhythm characteristics were significantly altered, by fasting (Figure 2a).

Intriguingly, the BAT clock was most responsive to the 24 h fast, with one or more changes in circadian rhythm characteristics in every core clock component tested (Figure 2a). Fasting caused a gain of rhythmicity in liver expression of *Cry2*, PWAT *Per2*, and BAT *Cry1*, *Cry2*, and *Rora* (Figure 2a). Notably, *Rora* was arrhythmic in fed and fasted conditions in all other tissues, while *Rorγ*, also highly expressed in the liver (Takeda et al., 2014), was strongly rhythmic. Fasting caused loss of rhythmicity in PWAT *Per1*, *Rev‐erbβ*, and *Cry2*, as well as BAT *Clock*.

Fasting increased the amplitude of *Bmal1* expression in BAT (peak: trough ratio of 12.7 and 17.9, *p* < 0.001), while it decreased in the liver (peak: trough ratio 35.9 to 5.8, *p* < 0.001). *Reverba* was dampened in the BAT (19 to 6, *p* < 0.01) and colon (19.9 to 9.8, *p* < 0.01). *Cry2* was slightly dampened in the duodenum (5.25 to 5, *p* = 0.05). The clock output gene *Dbp* was strongly dampened in the liver (*p* < 0.01), PWAT (*p* < 0.001), and duodenum (*p* < 0.01). The expression of *Clock* was arrhythmic in both conditions in the intestine (Figure 1g,h), and its rhythm was weak in brown and white adipose tissue (Figure 1e,f). Interestingly, *Per2* was arrhythmic in both conditions in the duodenum, but its mean expression was induced more than 3‐fold by fasting (Figure 1g).

### 3.2 Fasting alters clock‐controlled metabolic genes

The expression of metabolic genes known to be regulated by the core clock were also altered by fasting (Figure 2B). In the liver, the rate‐limiting enzyme of bile acid synthesis, cholesterol 7a‐hydroxylase *Cyp7a1*, was phase advanced (4.97 h, *p* < 0.01). Rhythmic expression of *Nampt*, the rate‐limiting enzyme of the NAD+ salvage pathway, was dampened. Expression of acetyl‐CoA carboxylase *Acc1*, encoding the first enzyme of de novo fatty acid synthesis, remained rhythmic during the fast but at significantly reduced mean levels (mesor decrease *p* < 0.0001) and amplitude (*p* < 0.01). In the PWAT, the master regulator of adipocyte differentiation and metabolism peroxisome proliferator‐activated receptor gamma *Pparγ* was phase advanced (5.6 h, *p* < 0.05). In the BAT, the expression of Angiopoietin‐like 4 *Angptl4*, which regulates lipoprotein lipase (LPL) activity and thereby fatty acid utilization in the BAT, was increased (mesor increase *p* < 0.0001) and phase delayed (8.53 h, *p* < 0.0001). Though each of these is known to be regulated by the core clock (Chen & Yang, 2014; Ferrell & Chiang, 2015; Lavery & Schibler, 1993; Nakahata et al., 2009; Ramsey et al., 2009; van den Berg et al., 2018), their alterations did not correlate with changes to core clock gene expression. Further studies are required to determine whether the core clock mediates these fasting‐induced shifts through other mechanisms, such as post‐translational modifications.

### 3.3 Microbiome rhythms are sustained in fasted mice

**FIGURE 3:**
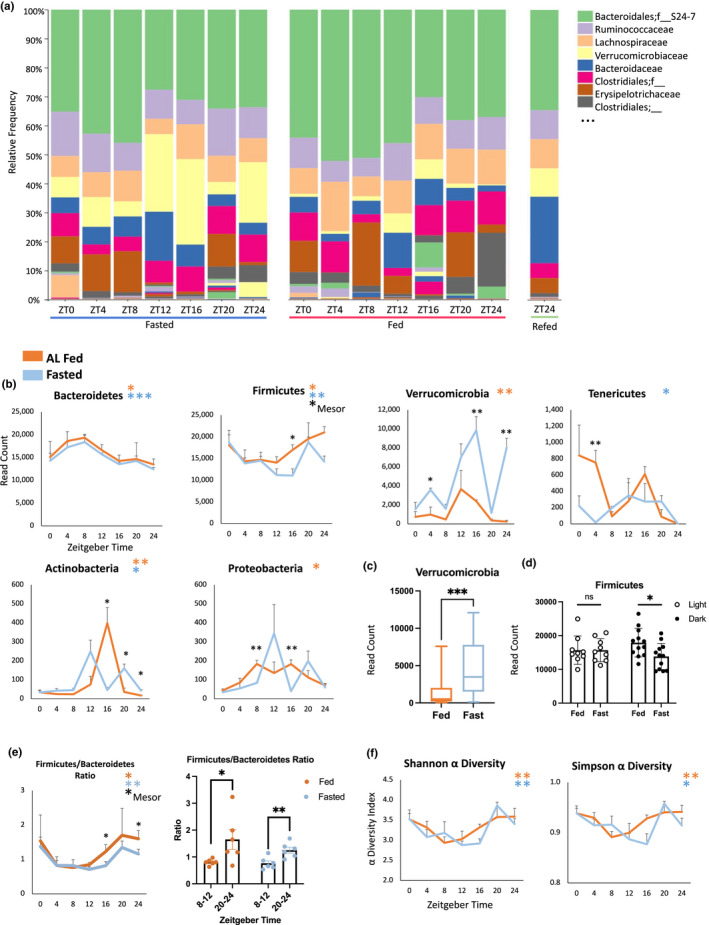
Rhythmicity of the microbiome. (a) Relative abundances of bacterial families. Bacteroidales and Bacteroidaceae belong to the phylum Bacteroidetes; Ruminococcaceae, Lachnospiraceae, Clostridiales, and Erysipelotrichaeceae belong to the phylum firmicutes; and Verrucomicrobiaceae belongs to the phylum Verrucomicrobia. A full list of families is available in Figure S3a. (b) Rhythms in rarefied abundances of the major bacterial phyla in AL fed (orange) and 24 h fasted (blue) mice. Black asterisks indicate significant differences between fed and fasted groups at a particular timepoint (TTEST), *n* = 3 mice/timepoint/group. Colored asterisks indicate presence of 24 h rhythm. *p < 0.05, **p < 0.01, ***p < 0.001. (c) Fasting increased mean expression levels of Verrucomicrobia. (d) Fasting depressed firmicutes during the dark phase. (e) Time dependence of the firmicutes/Bacteroidetes ratio. (f) Shannon and Simpson indices of α‐diversity, or within‐condition microbial diversity.

Unexpectedly, the relative abundances of bacteria continued to fluctuate diurnally in 24 h‐fasted mice (Figure 3a; Figure S3). Sequence read abundances were rarefied to account for differences in sequence counts between samples and analyzed using Circacompare (Parsons et al., 2020). The five most abundant bacterial phyla are Bacteroidetes, Firmicutes, Verrucomicrobia, Actinobacteria, and Proteobacteria (Arumugam et al., 2011). In both the fed and fasted condition, Bacteroidetes, Firmicutes, and Verrucomicrobia together comprised over 98% of detected microbes (Figure S3a). These three phyla were all rhythmic in the fed condition, and only Verrucomicrobia became arrhythmic in the fasted condition (Figure 3b). Proteobacteria was also arrhythmic in the fasted condition, whereas Tenericutes gained rhythmicity upon fasting (Figure 3b).

### 3.4 Fasting alters rhythmic abundances of bacterial phyla

The abundances of the major bacterial phyla are known to depend on the nutritional state, with the fed and fasted state being associated with Bacteroidetes and Firmicutes dominance, respectively. An increase in Bacteroidetes and decrease in Firmicutes was observed in healthy humans after a 10‐day Buchinger fast, a modified fasting regimen of ~200–250 kcal/day (de Toledo et al., 2013), and this was partially reversed following 4 days of food reintroduction (Mesnage et al., 2019). In mice, alternate day fasting increased Firmicutes and decreased Bacteroides abundance (Li et al., 2017), and a 48 h fast lead to dramatic increases in Verrucomicrobia (Miyamoto et al., 2019). Rhythms in feeding could thereby explain rhythms in bacterial abundance. AL‐fed mice consume the majority of calories during the active phase, at which time the proportional abundance of Firmicutes is highest, whereas Bacteroidetes and Verrucomicrobia are highest during the inactive, fasting phase of the daily cycle (Zarrinpar et al., 2014). Our results confirm a significant increase in Verrucomicrobia upon fasting throughout the 24 h cycle (Figure 3c). Interestingly, fasting decreased Firmicutes abundance as predicted, but only during the night (Figure 3d).

However, fasting had no effect on the rhythm of Bacteroidetes abundance (Figure 3b). The correlation between the natural fasting period of AL‐fed mice and the peak of Bacteroidetes (Zarrinpar et al., 2014) is therefore spurious. The Bacteroidetes rhythm is more robust (less variable) in the fasted condition and retains the same acrophase and amplitude as observed in AL fed mice. This suggests that daily fluctuations in Bacteroidetes are not the result of feeding rhythms, but rather the outcome of interaction with clock‐regulated processes in the host. The Bacteroidetes rhythm is also known to exhibit TRF effects, whereby restriction of feeding to the inactive phase causes phase inversion (Thaiss et al., 2014), and our results indicate that these are likely mediated by the entrainment of the host clock to feeding.

Bacteroidetes and Firmicutes comprise more than 90% of the entire microbial community in mice and humans, and an increased ratio of Firmicutes to Bacteroidetes is associated with obesity and metabolic disease (Turnbaugh et al., 2006). We found that the Firmicutes:Bacteroidetes ratio has a circadian rhythm in both the fed and fasted condition (Figure 3e). This rhythmicity results in a ratio that is approximately twice as high when measured at ZT20‐24 compared to ZT8‐12 (Figure 3e).

### 3.5 Microbial diversity is rhythmic in fed and fasted mice

Alpha diversity estimates the number of different taxa within a sample, or intra‐sample diversity. This can be estimated using Shannon and Simpson indices. Greater microbial diversity is associated with resilience and host health (Lozupone et al., 2012). We found that alpha diversity followed a circadian rhythm in both fed and fasted mice, with no difference in average levels of diversity over the course of the day between these groups (Figure 3f). The alpha diversity of the microbiota is known to fluctuate over time in mice with AL access to normal chow, rising during the night after food consumption compared to the daytime fast; this rhythm was lost in mice fed with HFD (Zarrinpar et al., 2014), which would seem to suggest that alpha diversity follows the food intake rhythm. However, our data indicate that microbial diversity retains its robust circadian rhythm in 24 h fasted animals, meaning that this rhythm depends on host circadian physiology rather than on food intake. Further supporting the role of the host circadian clock in producing rhythmic levels of alpha diversity, Clock^Δ19^‐mutant mice have lower diversity (Shannon and Simpson indices) compared to wildtype mice (Voigt et al., 2016).

### 3.6 Circadian rhythms at the family and genus levels

**FIGURE 4:**
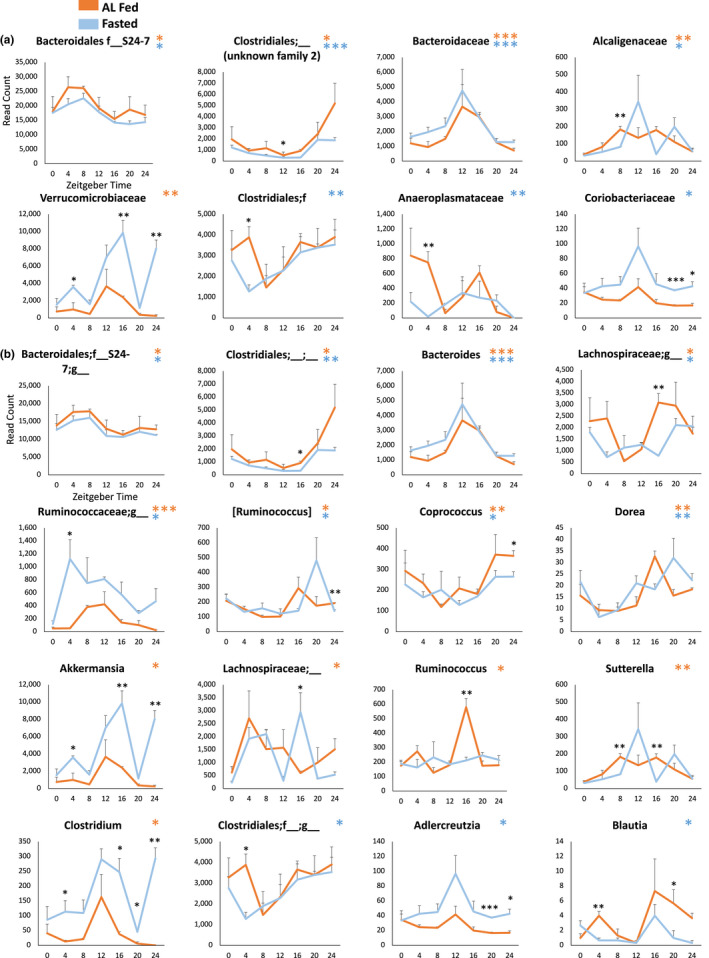
Sub‐phylum microbiome rhythm analysis. Circadian rhythms in rarefied absolute abundances of bacterial families (a) and genera (b) in AL fed (orange) and 24 h fasted (blue) mice. Black asterisks indicate significant differences between fed and fasted groups at a particular timepoint (TTEST), *n* = 3 mice/timepoint/group. Colored asterisks indicate significant rhythmicity. *p < 0.05, **p < 0.01, ***p < 0.001.

To further characterize the effect of fasting on rhythms in the microbiota, we performed sub‐phylum analyses, looking at bacterial families and genera (Figure 4a,b). A total of 28 families were detected, of which 13 were excluded from analysis due to insufficient reads (>15% of samples had zero reads). Of the remaining 15 families, four were rhythmic in the fed and fasted condition, one in the fed condition only, and three in the fasted condition only (Figure 4a; Table S2). A total of 46 genera were detected, of which 26 had sufficient reads. Of these 26 genera, eight were significantly rhythmic in both conditions, five only in fed mice, and three only in 24 h‐fasted mice (Figure 4B; Table S2). For all families and genera found to be rhythmic in both conditions, there were no significant changes in rhythm characteristics upon fasting.

### 3.7 Effects of time and feeding on microbial rhythmicity

**FIGURE 5:**
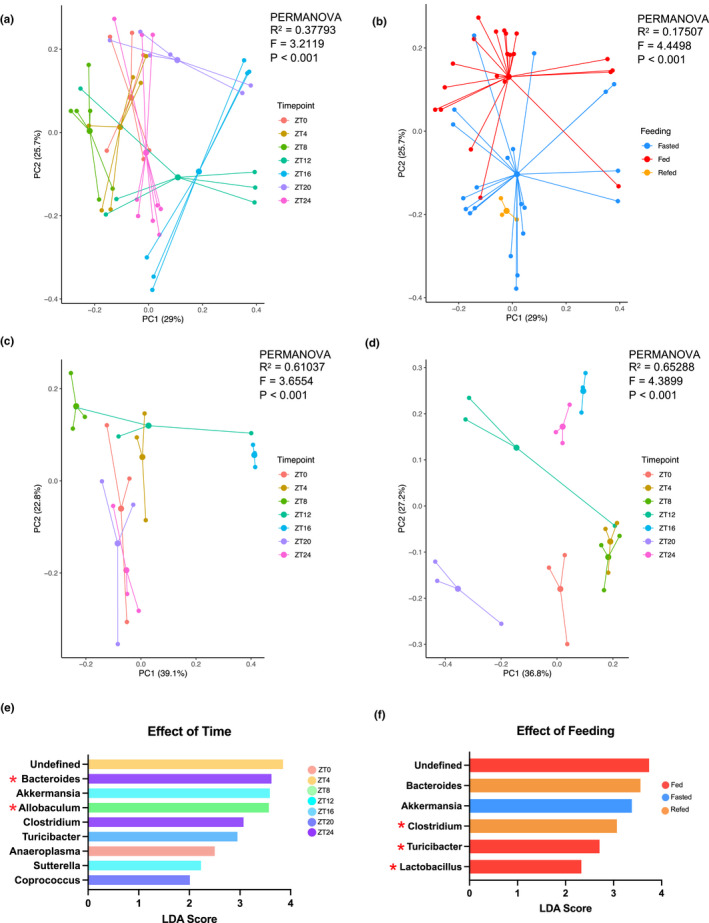
The effect of time of day and feeding versus fasting on microbiome composition. (a–d) PCoA plots of Bray‐Curtis dissimilarities. PERMANOVA was used to determine whether timepoint and feeding had a significant effect on microbiome composition. (a) the effect of time on microbiome composition. Data points are colored by timepoint (see legend). Color clustering by timepoint illustrates the 38% of variation explained by time. (b) the effect of feeding condition on microbiome composition. Data points are colored according to feeding group (see legend). 18% of variation was explained by feeding condition. (c) the effect of time in AL‐fed animals. (d) the effect of time in 24 h‐fasted animals. (E) Genera whose abundances significantly differed at particular timepoints (*p* < 0.05) with LDA scores >2 are shown. (f) Genera whose abundances significantly differed between feeding groups (*p* < 0.05) with LDA scored >2 are shown. Red stars indicate significant differences corroborated with ANCOM analysis.

A total of 386 OTUs were detected in the fed condition, and a distinct set of 384 OTUs were detected in the fasted condition. Comparing microbial composition, at the OTU or other taxonomic level, between samples provides estimates of beta diversity, which can be used with PERMANOVA to test for effects of particular variables (i.e., timepoint or feeding), or with principal coordinates analysis (PCoA) to visualize how samples differ based on the variables. At the OTU level, time had a greater influence on the variation in microbiota composition than did the feeding condition. The microbiome composition was similar moving through time from ZT0 to ZT8, then shifted at ZT12 and again at ZT20 before returning at ZT24 to align with ZT0 (Figure 5a). Nearly 38% of the variation in microbial composition could be explained by time point (*p* < 0.001, Figure 5a), and 18% of the variation in microbial composition could be explained by feeding (*p* < 0.001, Figure 5b). Though there is some overlap, the fed and fasted microbiomes differed, and that of mice refed 6 h was similar to the fasted group (Figure 5b). Time explained variation in the microbiota as much in the fasted as in the fed condition (*R* ^2^ = 0.65 and 0.61, respectively. Figure 5c,d). At the Family level, time again explained more of the microbial variation (41%, *p* < 0.0001) than did feeding condition (16%, *p* < 0.0001). However, the effect of time within the fasted condition was greater than within the fed (*R* ^2^ of 0.66 and 0.47, respectively. Figure S2).

### 3.8 Fasting increases metabolically protective *Akkermansia*

Linear Discriminant Analysis (LDA) Effect Size (LEfSe) analysis (Segata et al., 2011) was used to identify taxa that can discriminate between groups of samples by timepoint or feeding condition. Differential abundances were additionally verified using analysis of composition of microbiomes (ANCOM) (Mandal et al., 2015). Genera identified by both methods to significantly differ at particular timepoints were *Allobaculum* and *Bacteroides* (Figure 5e). The genera *Clostridium*, *Lactobacillus*, and *Turicibacter* distinguished the fed and fasted condition (Figure 5f). In the fed condition, *Turicibacter* and *Roseburia* additionally differentiated timepoints, as did *Turicibacter* and *Clostridium* in the fasted condition (Figure S2e,f).

Of the genera whose abundances significantly differed between feeding conditions, *Akkermansia* showed the highest effect size for increase by fasting (Figure 5f). It contributed on average 13.5% of reads to the fasted condition, compared to 3.6% in fed mice (*p* < 0.001). The upregulation of *Akkermansia* at ZT16 and ZT24 also appeared to drive a similar pattern in its family, Verrucomicrobia (Figure 4a). The role of *Akkermansia* as a beneficial microbe is well‐characterized (Naito et al., 2018). Abundances of species in this genus are inversely associated with metabolic disease, obesity, and inflammation (Zhou, 2017; Zhou et al., 2020), potentially through its effects on glucagon‐like peptide 1 (GLP‐1) secretion(Yoon et al., 2021).

## 4 DISCUSSION

The circadian system regulates whole body physiology in anticipation of regular daily cycles in the environment. In metabolic tissues, signals of feeding and fasting act as Zeitgebers which entrain the core clock. We demonstrate that core clock gene expression remains robustly rhythmic in 24 h fasted mice in the liver, PWAT, BAT, duodenum, and colon. This is the first time that the rhythmicity of peripheral tissue clocks has been extensively characterized in fed and fasted animals. While previous work demonstrated core clock gene rhythms in fed and 24 h‐fasted liver and skeletal muscle (Kinouchi et al., 2018), we contribute the rhythms of brown and white adipose tissue, intestine, and the microbiome, and further characterize the rhythms in all tissues using CircaCompare (Parsons et al., 2020). This method supports the comparison of rhythmic data, allowing a statistically robust demonstration of the differences in rhythm characteristics (Parsons et al., 2020).

We found that particular elements of the clock were altered in their amplitude, mean expression, and/or phase, while others lost or gained expression upon fasting, in a tissue‐specific manner. For example, fasting increased expression of *Per1* in the liver, *Per2* in the BAT, and *Cry2* in the duodenum. The tissue‐specific sensitivity of clock components to fasting suggests different mechanisms of circadian regulation by nutrient signaling. This is consistent with the variable speed at which key metabolic organs entrain to new food intake rhythms (Damiola et al., 2000). Future work identifying these mechanisms may allow targeted prevention of circadian misalignment in metabolism.

We found that of all metabolic tissues tested, the BAT was most sensitive to fasting. Significant changes to rhythmicity and rhythm parameters of amplitude, mesor, or phase were observed in every core clock gene. This was also the only tissue in which significant phase shifts were triggered by the 24 h fast; both *Per3* and *Rev‐erbβ* were phase delayed by approximately 5 h. Interestingly, the expression of *Angptl4* was greatly increased, and its peak was also phase delayed (approximately 8.5 h). The role of *Angptl4* in the BAT is to inhibit lipoprotein lipase (LPL) activity, thereby reducing free fatty acid (FFA) uptake. During cold exposure, it is downregulated to permit thermogenesis through fatty acid combustion by the BAT (Dijk et al., 2015). This is important because the BAT acts as a metabolic sink, taking up FFA in a time‐of‐day dependent manner that affects postprandial lipid clearance (van den Berg et al., 2018) and systemic energy availability. Our findings suggest that the circadian clock may be involved in inhibiting FFA utilization by BAT during fasting, which would contribute to a decreased energy expenditure (Levin & Trayhurn, 1987) and conserve FFA for essential organs. At the same time, this would be expected to reduce non‐shivering thermogenesis, exposing the animal to potential hypothermia.

A second major finding is that circadian rhythms in the microbiome are sustained in fasted animals. The alpha diversity of the microbiome was also rhythmic and sustained during fasting. The two major bacterial phyla, Bacteroidetes and Firmicutes, displayed even more robust 24 h rhythmicity in the fasted compared to the fed condition. Nutrient intake rhythms are therefore not the sole driver of circadian variation in the microbiota. In a complementary study, microbial abundance continued to vary diurnally in mice given continuous IV parenteral nutrition for 72 h (Leone et al., 2015). Together these results strongly implicate the endogenous clock of the host in the regulation of rhythmic bacterial abundance. An intact circadian system is sufficient to drive rhythms in the gut microbiota independent of food intake rhythms.

The necessity of clock genes in the host for robust microbial rhythms has been shown in whole‐body knockouts of both the positive [*Bmal1* (Liang et al., 2015)] and negative [*Per1/2* (Thaiss et al., 2014, 2016)] arms of the core clock. However, the effects of clock disruption are confounded by arrhythmic activity and feeding (Butler & Gibbs, 2020). Feeding a high fat diet, which is known to cause disorganized feeding rhythms (Kohsaka et al., 2007), also caused dampened gut microbial rhythms in mice (Leone et al., 2015; Zarrinpar et al., 2014). Time‐restricted feeding (TRF) to the active phase partially restored microbial rhythms in HFD‐fed animals (Zarrinpar et al., 2014) and Per1/2 knockouts (Thaiss et al., 2014). This may seem to suggest that feeding rhythms control microbial rhythms. However, TRF also entrains endogenous rhythms in host tissues, leaving it unclear whether TRF restores microbial rhythms directly through nutrient availability, or indirectly through effects on host tissue clocks (Liang et al., 2015). Our results support the latter hypothesis.

The relationships between the microbiome and host circadian system are multifaceted and bidirectional (Bishehsari et al., 2020). Microbial interactions with intestinal epithelial cells are essential for their clock function (Mukherji et al., 2013) and impact expression of nutrient transporters and metabolic genes (Thaiss et al., 2016; Wang et al., 2018). Moreover, metabolite oscillations in the gut are reflected in the serum, and abolished in germ free or antibiotic‐treated mice (Thaiss et al., 2016). The microbiome thereby has distal effects on key metabolic organs; the short chain fatty acid (SCFA) butyrate modulates the core clock in the liver (Leone et al., 2015), and the microbiome mediates PPARy‐driven metabolic reprogramming in the liver upon high‐fat feeding (Murakami et al., 2016). Fluctuations in microbial abundance therefore contribute to the rhythmicity of nutrient absorption and systemic metabolism in the host, and our results indicate this component of circadian physiology persists also in the fasted condition.

Though direct relationships cannot be inferred, it is interesting to note that the robust rhythm of Bacteroidetes abundance has a clear antiphase relationship to the colonic expression of *Bmal1*. Similarly, Firmicutes abundance appears to be in phase with colonic *Bmal1* expression. This could suggest the influence of the negative and positive arms of the core clock on these phyla, respectively. Further studies are needed to investigate the relationship between the core clock in the colon and rhythms in the microbiome. Future studies would benefit from the use of wire‐bottomed cages and limited bedding during the fasting period to control for coprophagia and fiber consumption from cage bedding (Gregor et al., 2020). A related limitation of the present study is that mice from each timepoint and feeding condition were cohoused in the days leading up to feces collection, which can produce cage effects in the microbiome (Ericsson et al., 2018). Nonetheless, there was substantial variation in the microbiome composition of mice within a cage (Figure S3b), and the significant circadian rhythmicity, wherein many taxa closely fit a 24 h cosinar function, supports that this variation is physiological rather than artefactual.

The present study emphasizes that the recency of feeding and the time of sample collection are two essential parameters to consider when assessing not only circadian physiology, but also the expression of important metabolic genes or the composition of the microbiome. These vary greatly on the scale of just a few hours. For example, the Firmicutes:Bacteroidetes ratio has a circadian rhythm; it is approximately twice as high when measured at the end of the dark phase (ZT20‐24) as compared to the end of the light phase (ZT8‐12). In studies using this ratio as a metric of metabolic health (Turnbaugh et al., 2006), it would be possible to observe significant changes simply as a result of microbial samples from two groups being collected at different times of day. When considering study design optimization, timed and repeated sampling from individual subjects, rather than single samples from the maximal number of subjects, may produce more consistent and accurate results (Poyet et al., 2019; Vázquez‐Baeza et al., 2018). Future investigations of metabolic organs and the microbiome will benefit from a circadian‐informed design.

In conclusion, our study characterizes the effect of an acute 24 h fast on circadian gene expression in key metabolic organs, including for the first time the intestine and brown adipose tissue (BAT). Core clock rhythmicity remains robust in the fasted state, with tissue‐specific changes to rhythm parameters, particularly in the BAT. Examples of large changes in the amplitude and phase of key metabolic genes across the 24 h period and in the fed versus fasted condition emphasize the importance of these parameters in study design. We further show that the abundances of bacterial phyla in the fecal microbiota are rhythmic. Phyla‐specific alterations were observed upon fasting, however, bacterial abundances retained significant 24 h rhythmicity even in the absence of rhythmic nutrient availability. This strongly implicates the endogenous host circadian system in the regulation of daily fluctuations of the microbiota.

## AUTHOR CONTRIBUTIONS

L.P., H.K.S, J.H.L., and N.V. designed the experiments. L.P., J.H.L., N.V., Q.W.S., and C.Y. conducted the experiments. D.G. performed 16S rRNA sequencing. H.M. performed statistical analysis of microbiome data. L.P. and H.K.S. wrote the manuscript.

## CONFLICT OF INTEREST

The authors declare no competing interests.

## Supporting information


**Appendix S1** Supporting Information
