# Supplementary material for: Circadian rhythms in metabolic organs and the microbiota during acute fasting in mice
Source: Physiol Rep. 2022 Jul 19;10(14):e15393. doi: 10.14814/phy2.15393 (PMC9295129; doi:10.14814/phy2.15393)
Supplement: Supplementary file 1 — Appendix S1 Supporting Information [file PHY2-10-e15393-s001.pdf]

Supplementary Figure S1

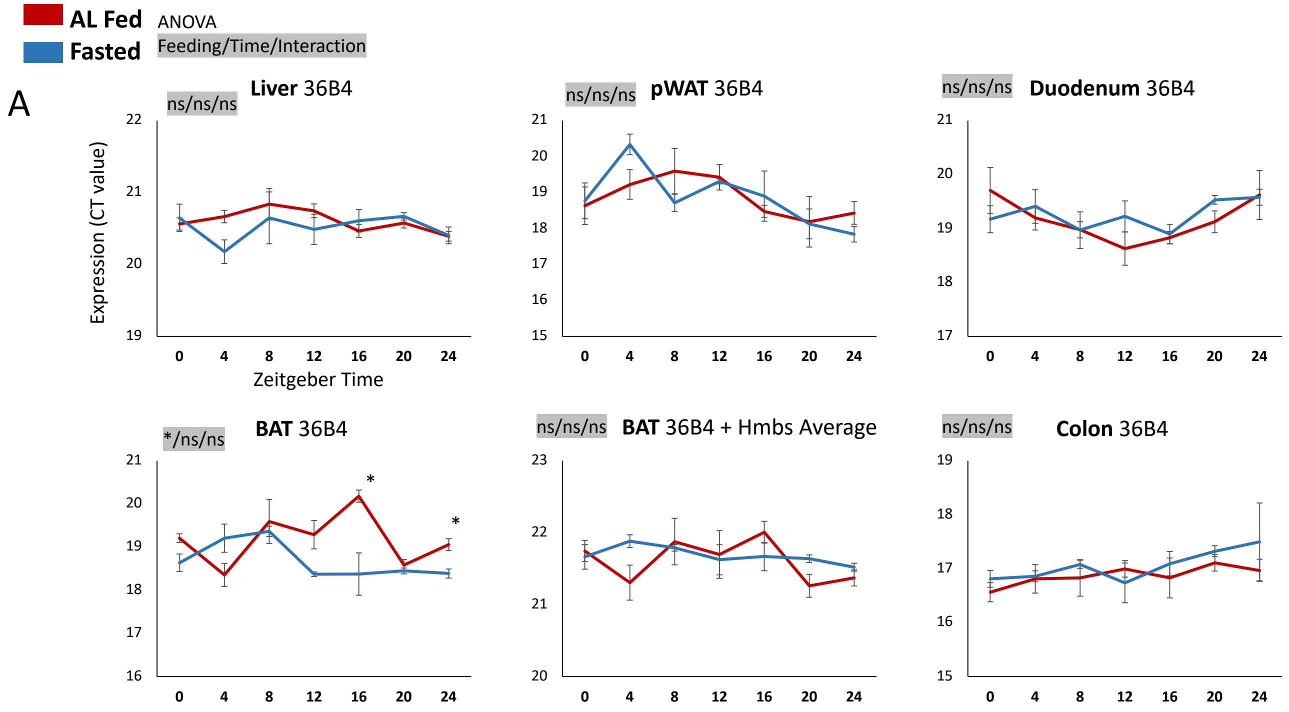

B

CIRCADIAN		ANOVA		
**Tissue**	**Gene**	**Feeding**	**Time**	**Interaction**
Liver	Bmal1	p=.05	***	***
Liver	Clock	ns	**	ns
Liver	Dbp	ns	**	***
Liver	Per1	ns	p=.06	*
Liver	Per2	ns	*	**
Liver	Per3	ns	***	***
Liver	Cry1	ns	**	**
Liver	Cry2	ns	***	ns
Liver	Rev-ebα	**	ns	ns
Liver	Rev-erbβ	ns	*	**
Liver	Rorα	ns	**	***
Liver	Rorγ	*	**	***
PWAT	Bmal1	*	*	***
PWAT	Clock	ns	ns	ns
PWAT	Dbp	ns	*	***
PWAT	Per1	ns	ns	*
PWAT	Per2	ns	**	*
PWAT	Per3	ns	***	**
PWAT	Cry1	ns	ns	**
PWAT	Cry2	ns	***	ns
PWAT	Rev-ebα	ns	ns	*
PWAT	Rev-erbβ	ns	**	*
PWAT	Rorα	ns	ns	ns
BAT	Bmal1	**	*	***
BAT	Clock	ns	p=.05	ns
BAT	Dbp	ns	**	***
BAT	Per1	ns	p=.065	**
BAT	Per2	***	ns	**
BAT	Per3	ns	p=.06	**
BAT	Cry1	*	ns	**
BAT	Cry2	ns	*	ns
BAT	Rev-ebα	ns	p=.06	*
BAT	Rev-erbβ	ns	*	**
BAT	Rorα	ns	ns	**

CIRCADIAN		ANOVA		
**Tissue**	**Gene**	**Feeding**	**Time**	**Interaction**
Duodenum	Bmal1	ns	***	ns
Duodenum	Clock	*	ns	ns
Duodenum	Dbp	ns	***	ns
Duodenum	Per1	ns	***	ns
Duodenum	Per2	p=.07	ns	ns
Duodenum	Per3	ns	***	ns
Duodenum	Cry1	ns	***	ns
Duodenum	Cry2	ns	**	ns
Duodenum	Rev-ebα	ns	ns	ns
Duodenum	Rev-erbβ	ns	***	ns
Duodenum	Rorα	ns	***	p=.06
Colon	Bmal1	ns	***	ns
Colon	Clock	ns	ns	ns
Colon	Dbp	ns	***	ns
Colon	Per1	ns	*	n**
Colon	Per2	*	***	ns
Colon	Per3	ns	***	*
Colon	Cry1	ns	**	ns
Colon	Cry2	ns	ns	ns
Colon	Rev-ebα	ns	***	ns
Colon	Rev-erbβ	ns	***	ns
Colon	Rorα	ns	ns	*

OTHER		ANOVA		
**Tissue**	**Gene**	**Feeding**	**Time**	**Interaction**
Liver	Cyp7a1	*	***	*
Liver	SREBP1c	ns	ns	***
Liver	Nampt	ns	**	**
Liver	Fgf21	ns	ns	**
Liver	Acc1	ns	ns	***
PWAT	PPARg	ns	**	ns
BAT	Angptl4	*	***	***
Duodenum	HMGCS2	**	ns	*

Supplementary Figure S2

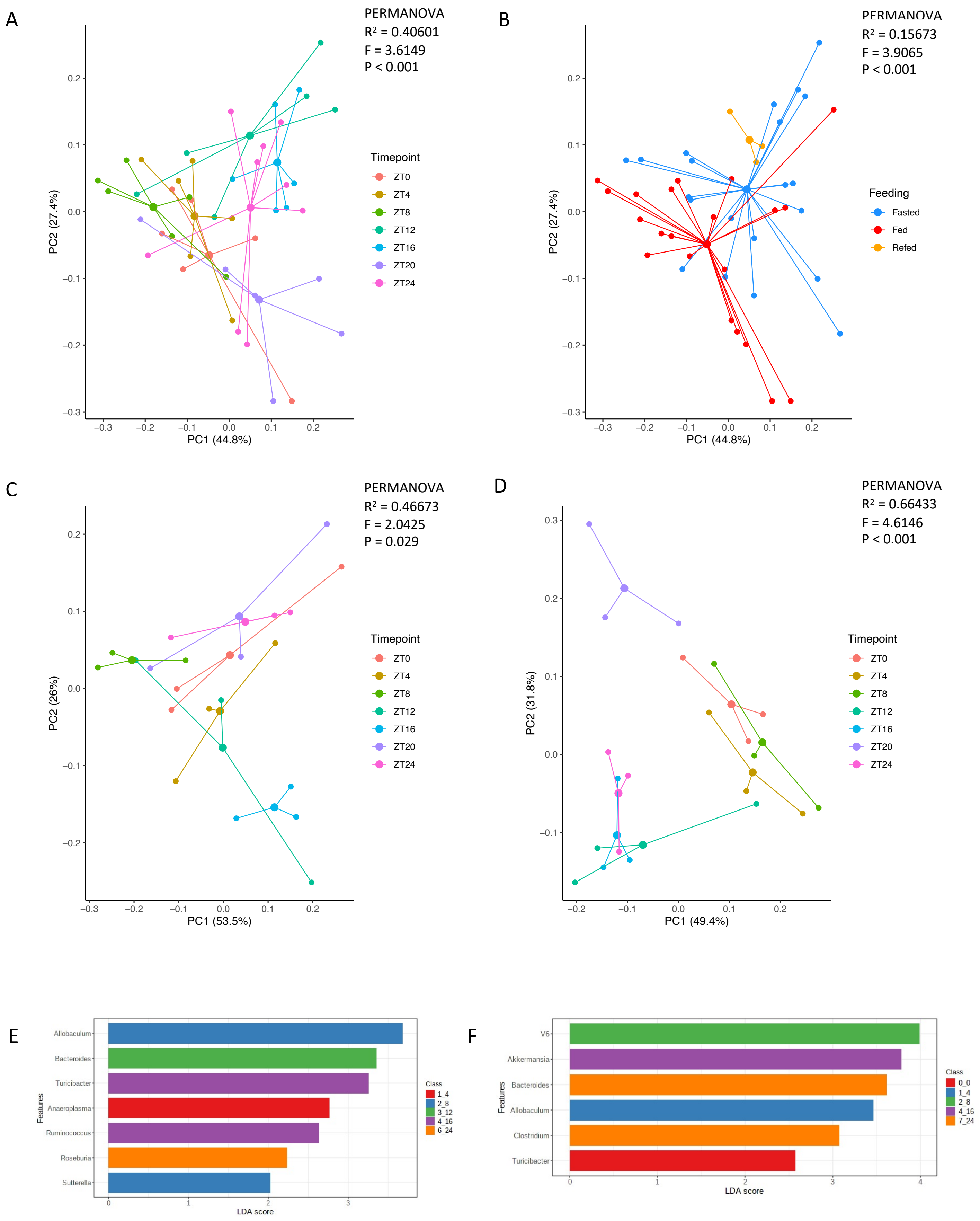

Supplementary Figure S3

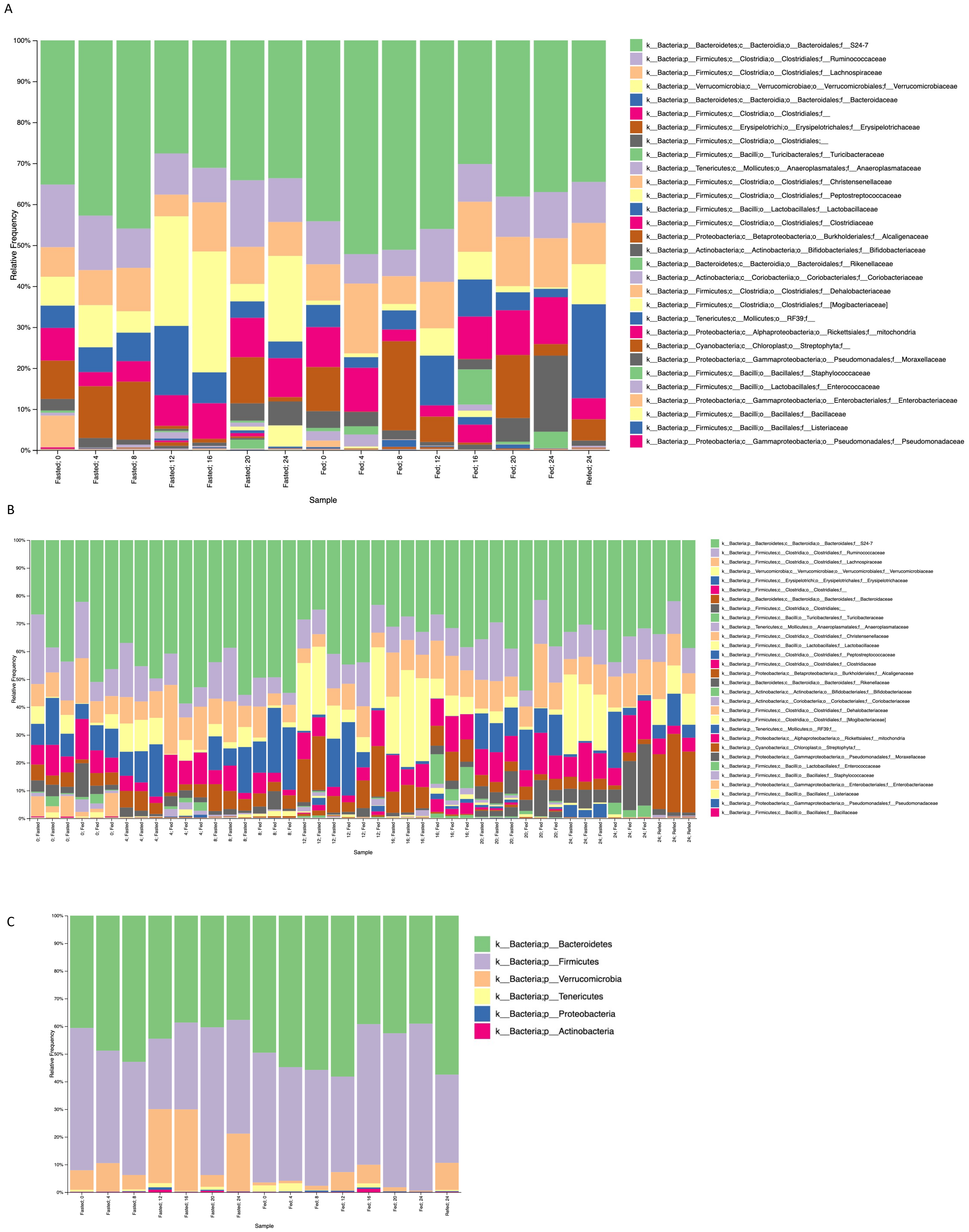

Supplementary Table S1

**CIRCADIAN**		
**Target**	**Forward**	**Reverse**
Bmal1	CCTAATTCTCAGGGCAGCAGAT	TCCAGTCTTGGCATCAATGAGT
Clock	GAGGTCGTCCTTCAGCAGTC	TGTGACATGCCTTGTGGAAT
Cry1	GACTCACTCACTCAAGCAAG	TCCTCCCACACGCTTTCGTATC
Cry2	ATGTGTTCCCAAGGCTGTTC	CCTCCTTGGCCATCTTCATA
Dbp	AATGACCTTTGAACCTGATCCCGCT	GCTCCAGTACTTCTCATCCTTCTGT
Per1	TGGCTCAAGTGGCAATGAGTC	GGCTCGAGCTGACTGTTCACT
Per2	CCATCCACAAGAAGATCCTAC	GCTCCACGGGTTGATGAAGC
Per3	GTGACAGCAGAGTCCCATGA	CACTGCCATCTCGAGTTCAA
Rev-erba	ACGACCCTGGACTCCAATAA	CCATTGGAGCTGTCACTGTAGA
Rev-erbb	ACAGAAATAGTTACCTGTGCAACACT	GACTTGCTCATAGGACACACCA
Ror-a	CCCCTACTGTTCCTTCACCA	CCAGGTGGGATTTGGATATG
Ror-g	ACTACGGGGTTATCACCTGTGAG	GTGCAGGAGTAGGCCACATTAC
**OTHER**		
**Target**	**Forward**	**Reverse**
36B4	CAGATGCAGCAGATCCGCA	CGCATCATGGTGTTCTTGCC
Hmbs	AAGGGTTTTCCCGTTTGC	TCCCTGAAGGATGTGCCTA
Cyp7a1	AGCAACTAAACAACCTGCCAGTACTA	GTCCGGATATTCAAGGATGCA
SREBP1c	GGAGCCATGGATTGCACATT	GGCCCGGGAAGTCACTGT
Nampt	TCGGTTCTGGTGGCGCTTTGCTAC	AAGTTCCCCGCTGGTGTCCTATGT
Fgf21	CGGCCCTGTAAAGGCTCT	CGGCCCTGTAAAGGCTCT
Acc1	GTTCTGTTGGACAACGCCTTCAC	GGAGTCACAGAAGCAGCCCATT
PPARg	CTGCTCAAGTATGGTGTCCATGA	TGAGATGAGGACTCCATCTTTATTCA
Angptl4	GGCTCAGAACAGCAAGATCC	CCTCTTTCCCCTCGAAGTCT
HMGCS2	CAATGTCACCACAGACCACCAG	CAATGTCACCACAGACCACCAG

Supplementary Table S2

Phylum	Rhythmic
k__Bacteria;p__Bacteroidetes	Yes
k__Bacteria;p__Firmicutes	Yes
k__Bacteria;p__Proteobacteria	Fed only
k__Bacteria;p__Actinobacteria	Yes
k__Bacteria;p__Verrucomicrobia	Fed only
k__Bacteria;p__Tenericutes	Fast only
k__Bacteria;p__Cyanobacteria	insufficient counts
**Family**	
k__Bacteria;p__Bacteroidetes;c__Bacteroidia;o__Bacteroidales;f__S24-7	Yes
k__Bacteria;p__Firmicutes;c__Clostridia;o__Clostridiales;f__Ruminococcaceae	No
k__Bacteria;p__Firmicutes;c__Clostridia;o__Clostridiales;f__	Fast only
k__Bacteria;p__Firmicutes;c__Clostridia;o__Clostridiales;f__Lachnospiraceae	No
k__Bacteria;p__Firmicutes;c__Clostridia;o__Clostridiales;__	Yes
k__Bacteria;p__Firmicutes;c__Clostridia;o__Clostridiales;f__Clostridiaceae	insufficient counts
k__Bacteria;p__Firmicutes;c__Erysipelotrichi;o__Erysipelotrichales;f__Erysipelotrichaceae	No
k__Bacteria;p__Firmicutes;c__Clostridia;o__Clostridiales;f__Dehalobacteriaceae	No
k__Bacteria;p__Firmicutes;c__Bacilli;o__Turicibacterales;f__Turicibacteraceae	insufficient counts
k__Bacteria;p__Firmicutes;c__Clostridia;o__Clostridiales;f__Christensenellaceae	insufficient counts
k__Bacteria;p__Proteobacteria;c__Betaproteobacteria;o__Burkholderiales;f__Alcaligenaceae	Yes
k__Bacteria;p__Firmicutes;c__Clostridia;o__Clostridiales;f__Peptostreptococcaceae	insufficient counts
k__Bacteria;p__Actinobacteria;c__Coriobacteriia;o__Coriobacteriales;f__Coriobacteriaceae	Fast only
k__Bacteria;p__Verrucomicrobia;c__Verrucomicrobiae;o__Verrucomicrobiales;f__Verrucomicrobiaceae	Fed only
k__Bacteria;p__Tenericutes;c__Mollicutes;o__Anaeroplasmatales;f__Anaeroplasmataceae	Fast only
k__Bacteria;p__Firmicutes;c__Clostridia;o__Clostridiales;f__[Mogibacteriaceae]	No
k__Bacteria;p__Tenericutes;c__Mollicutes;o__RF39;f__	insufficient counts
k__Bacteria;p__Firmicutes;c__Bacilli;o__Lactobacillales;f__Lactobacillaceae	No
k__Bacteria;p__Proteobacteria;c__Alphaproteobacteria;o__Rickettsiales;f__mitochondria	insufficient counts
k__Bacteria;p__Proteobacteria;c__Gammaproteobacteria;o__Pseudomonadales;f__Moraxellaceae	insufficient counts
k__Bacteria;p__Cyanobacteria;c__Chloroplast;o__Streptophyta;f__	insufficient counts
k__Bacteria;p__Bacteroidetes;c__Bacteroidia;o__Bacteroidales;f__Bacteroidaceae	Yes
k__Bacteria;p__Firmicutes;c__Bacilli;o__Bacillales;f__Listeriaceae	insufficient counts
k__Bacteria;p__Firmicutes;c__Bacilli;o__Bacillales;f__Staphylococcaceae	insufficient counts
k__Bacteria;p__Firmicutes;c__Bacilli;o__Lactobacillales;f__Enterococcaceae	insufficient counts
k__Bacteria;p__Bacteroidetes;c__Bacteroidia;o__Bacteroidales;f__Rikenellaceae	insufficient counts
k__Bacteria;p__Proteobacteria;c__Gammaproteobacteria;o__Enterobacteriales;f__Enterobacteriaceae	insufficient counts
k__Bacteria;p__Actinobacteria;c__Actinobacteria;o__Bifidobacteriales;f__Bifidobacteriaceae	insufficient counts
**Genus**	
k__Bacteria;p__Bacteroidetes;c__Bacteroidia;o__Bacteroidales;f__S24-7;g__	Yes
k__Bacteria;p__Firmicutes;c__Clostridia;o__Clostridiales;f__Ruminococcaceae;g__Oscillospira	No
k__Bacteria;p__Firmicutes;c__Clostridia;o__Clostridiales;f__Ruminococcaceae;g__Ruminococcus	Yes
k__Bacteria;p__Firmicutes;c__Clostridia;o__Clostridiales;f__;g__	Fast only
k__Bacteria;p__Firmicutes;c__Clostridia;o__Clostridiales;f__Lachnospiraceae;g__Coprococcus	Yes
k__Bacteria;p__Firmicutes;c__Clostridia;o__Clostridiales;f__Lachnospiraceae;g__	Yes
k__Bacteria;p__Firmicutes;c__Clostridia;o__Clostridiales;f__Lachnospiraceae;__	Fed only
k__Bacteria;p__Firmicutes;c__Clostridia;o__Clostridiales;f__Ruminococcaceae;g__	Yes
k__Bacteria;p__Firmicutes;c__Clostridia;o__Clostridiales;__;__	Yes
k__Bacteria;p__Firmicutes;c__Clostridia;o__Clostridiales;f__Ruminococcaceae;__	No
k__Bacteria;p__Firmicutes;c__Clostridia;o__Clostridiales;f__Clostridiaceae;__	insufficient counts
k__Bacteria;p__Firmicutes;c__Clostridia;o__Clostridiales;f__Lachnospiraceae;g__Roseburia	insufficient counts
k__Bacteria;p__Firmicutes;c__Erysipelotrichi;o__Erysipelotrichales;f__Erysipelotrichaceae;g__Clostridium	Fed only
k__Bacteria;p__Firmicutes;c__Clostridia;o__Clostridiales;f__Dehalobacteriaceae;g__Dehalobacterium	No
k__Bacteria;p__Firmicutes;c__Clostridia;o__Clostridiales;f__Lachnospiraceae;g__[Ruminococcus]	Fed only
k__Bacteria;p__Firmicutes;c__Bacilli;o__Turicibacterales;f__Turicibacteraceae;g__Turicibacter	insufficient counts
k__Bacteria;p__Firmicutes;c__Clostridia;o__Clostridiales;f__Christensenellaceae;g__	insufficient counts
k__Bacteria;p__Proteobacteria;c__Betaproteobacteria;o__Burkholderiales;f__Alcaligenaceae;g__Sutterella	Fed only
k__Bacteria;p__Firmicutes;c__Clostridia;o__Clostridiales;f__Peptostreptococcaceae;g__	insufficient counts
k__Bacteria;p__Actinobacteria;c__Coriobacteriia;o__Coriobacteriales;f__Coriobacteriaceae;g__Adlercreutzia	Fast only
k__Bacteria;p__Verrucomicrobia;c__Verrucomicrobiae;o__Verrucomicrobiales;f__Verrucomicrobiaceae;g__Akkermansia	Fed only
k__Bacteria;p__Firmicutes;c__Erysipelotrichi;o__Erysipelotrichales;f__Erysipelotrichaceae;g__Allobaculum	No
k__Bacteria;p__Tenericutes;c__Mollicutes;o__Anaeroplasmatales;f__Anaeroplasmataceae;g__Anaeroplasma	insufficient counts
k__Bacteria;p__Firmicutes;c__Clostridia;o__Clostridiales;f__[Mogibacteriaceae];g__	No

Supplementary Table S2 continued

[illegible]	[illegible]
k__Bacteria;p__Firmicutes;c__Clostridia;o__Clostridiales;f__Lachnospiraceae;g__Dorea	Yes
k__Bacteria;p__Firmicutes;c__Erysipelotrichi;o__Erysipelotrichales;f__Erysipelotrichaceae;g__	insufficient counts
k__Bacteria;p__Firmicutes;c__Clostridia;o__Clostridiales;f__Ruminococcaceae;g__Butyricicoccus	No
k__Bacteria;p__Tenericutes;c__Mollicutes;o__RF39;f__;g__	insufficient counts
k__Bacteria;p__Firmicutes;c__Bacilli;o__Lactobacillales;f__Lactobacillaceae;g__Lactobacillus	No
k__Bacteria;p__Firmicutes;c__Clostridia;o__Clostridiales;f__Lachnospiraceae;g__Blautia	Fast only
k__Bacteria;p__Actinobacteria;c__Coriobacteriia;o__Coriobacteriales;f__Coriobacteriaceae;g__	insufficient counts
k__Bacteria;p__Proteobacteria;c__Alphaproteobacteria;o__Rickettsiales;f__mitochondria;__	insufficient counts
k__Bacteria;p__Firmicutes;c__Clostridia;o__Clostridiales;f__Ruminococcaceae;g__Clostridium	insufficient counts
k__Bacteria;p__Firmicutes;c__Clostridia;o__Clostridiales;f__Lachnospiraceae;g__Clostridium	insufficient counts
k__Bacteria;p__Firmicutes;c__Clostridia;o__Clostridiales;f__Clostridiaceae;g__Clostridium	insufficient counts
k__Bacteria;p__Firmicutes;c__Erysipelotrichi;o__Erysipelotrichales;f__Erysipelotrichaceae;g__Coprobacillus	insufficient counts
k__Bacteria;p__Proteobacteria;c__Gammaproteobacteria;o__Pseudomonadales;f__Moraxellaceae;g__Acinetobacter	insufficient counts
k__Bacteria;p__Cyanobacteria;c__Chloroplast;o__Streptophyta;f__;g__	insufficient counts
k__Bacteria;p__Bacteroidetes;c__Bacteroidia;o__Bacteroidales;f__Bacteroidaceae;g__Bacteroides	Yes
k__Bacteria;p__Firmicutes;c__Bacilli;o__Bacillales;f__Listeriaceae;g__Listeria	insufficient counts
k__Bacteria;p__Firmicutes;c__Bacilli;o__Bacillales;f__Staphylococcaceae;g__Staphylococcus	insufficient counts
k__Bacteria;p__Firmicutes;c__Bacilli;o__Lactobacillales;f__Enterococcaceae;g__Enterococcus	insufficient counts
k__Bacteria;p__Bacteroidetes;c__Bacteroidia;o__Bacteroidales;f__Rikenellaceae;g__	insufficient counts
k__Bacteria;p__Proteobacteria;c__Gammaproteobacteria;o__Enterobacteriales;f__Enterobacteriaceae;__	insufficient counts
k__Bacteria;p__Firmicutes;c__Clostridia;o__Clostridiales;f__Lachnospiraceae;g__Anaerostipes	No
k__Bacteria;p__Actinobacteria;c__Actinobacteria;o__Bifidobacteriales;f__Bifidobacteriaceae;g__Bifidobacterium	insufficient counts

Figure S1
(A) Housekeeping gene expression. Upper left asterisks indicate results of 2-way repeated measures ANOVA: feeding/time/interaction. All are arrhythmic in the fed and fasted condition.
(B) Results of 2-way repeated measures ANOVA for feeding, time, and their interaction in all gene expression rhythms. * $p < 0.05$, ** $p < 0.01$, *** $p < 0.001$.

Figure S2
(A, B) Family level PCoA plots of Bray-Curtis dissimilarities. PERMANOVA was used to determine whether timepoint and feeding had significant effects on microbiome composition.
(A) The effect of time on microbiome composition. Data points are labelled according to feeding group (F = Fed, X = Fast, R = Refed) and coloured by timepoint (see legend).
(B) The effect of feeding condition on microbiome composition. Data points are labelled with timepoint and coloured according to feeding group (see legend).
(C) The effect of time in AL-fed animals.
(D) The effect of time in 24h-fasted animals.
(E) Families whose abundances significantly differed at in the fed condition at particular timepoints ($p < 0.05$) with LDA scores >2.
(F) Families whose abundances significantly differed at in the fasted condition at particular timepoints ($p < 0.05$) with LDA scores >2.

Figure S3
(A) Relative abundances of bacterial families by group.
(B) Relative abundances of bacterial families in individual mice. Mice of the same feeding condition and timepoint were cohoused.
(C) Relative abundances of bacterial phyla by group.

Table S1
qPCR primers

Table S2
Rhythmicity of microbial phyla, families and genera determined from CircaCompare analysis of rarefied read counts.
